# Prevalence and factors associated with preterm birth in a rural district hospital, Rwanda

**DOI:** 10.11604/pamj.2022.43.173.34113

**Published:** 2022-12-05

**Authors:** Hellen Chinwe Nwankwo, Michael Habtu, Erigene Rutayisire, Richard Kalisa

**Affiliations:** 1 Mount Kenya University, School of Health Sciences, Department of Public Health, Kigali Campus, Rwanda,; 2 School of Public Health, University of Rwanda, Kigali, Rwanda

**Keywords:** Antenatal care, maternal nutrition, preterm birth, preterm delivery

## Abstract

**Introduction:**

globally, the leading cause of neonatal mortality is preterm birth which may hinder the achievement of Sustainable Development Goal 3.2 target. We aimed to determine the prevalence and factors associated with preterm delivery at Kabutare hospital, Rwanda.

**Methods:**

a cross-sectional study was conducted between August and September 2020. Mothers were interviewed using a standard pretested semi-structured questionnaire and additional data were extracted from medical records of obstetric files. Gestational age was assessed using the Ballard score. Adjusted Odds Ratios and their 95% confidence intervals were calculated for multivariable logistic regression analysis to take care of all potential confounders.

**Results:**

the prevalence of preterm birth was 17.5% (95% CI: 12.9% - 22.9%). The independent factors associated with preterm birth after considering multiple logistic regression were husband being a smoker (adjusted Odds Ratio (aOR) = 5.9; 95% CI; 1.9-18; p= 0.002), antenatal care (ANC) attendance ≤ 3 visits (aOR=3.9; 95% CI; 1.1-13.8; p=0.04) and low mother's Mid Upper Arm Circumference (MUAC) < 23cm (aOR=5.6, 95% CI; 1.8-18.9; p=0.004). **Conclusion:** preterm delivery was high in Huye district. Thus, we recommend ANC sessions to emphasize on maternal nutritional education which is of good quality and quantity, discourage maternal alcohol consumption as well as passive smoking.

## Introduction

Preterm birth is the onset of delivery before 37 weeks of gestation, or lesser than 259 days from the last menstrual cycle [1]. It is one of the leading causes of neonatal morbidity and mortality and if left unattended, to achieve the Sustainable Development Goal 3 target of reaching the neonatal mortality rate to 12 per 1000 live birth by 2030 will be impossible [2]. Globally, 15 million preterm births are born annually, and of these births 81% occur within Sub-Sahara Africa and Asia [1]. Although most low and middle countries have inadequate data on preterm birth, where it has been documented in developed countries preterm birth rates has generally been on the rise over the past two decades [3]. Preterm birth may result from compound mixture of medical, biological, genetic, psychosocial, and situational environments. Approximately two thirds of the risk factors are unidentified; however, one third arises from clinical reference for a wide-scope of mother's and fetal diseases [4]. Maternal/obstetric risk factors include pregnancy at a younger age, short periods between births, inadequate prenatal visits, nutrition, and life style (alcohol, tobacco, smoking, prohibited drugs use) and medical problems (chronic hypertension, hypertensive disorders of gestation, diabetes mellitus, anemic mothers, obesity, maternal HIV status, sexually transmitted infections among others) are known risks factors [1,3-6]

In Rwanda, 43,000 babies were born too soon each year and 2,070 children under five die due to direct preterm complications [7]. Of recent, Rwanda reported a slight decline in neonatal mortality rate from 20 per 1,000 livebirth in 2015 to 19 per 1,000 live in 2020, which latter contributed to the same increase in infant mortality rate from 32 to 33 per livebirth [8,9]. Thus, identifying and understanding the factors associated with preterm birth has the potential to help address this burden. It is against this backdrop that this study sought to identify factors associated with preterm birth in Huye district so as to come up with ways of improving this burden.

## Methods

**Study design and setting:** this was a facility based cross-sectional study done among preterm babies delivered at Kabutare hospital, Huye district, Rwanda. Study variables included maternal socio-demographic characteristics, pregnancy lifestyle factors by preterm birth, antenatal and obstetric factors associated with preterm birth. Kabutare hospital is a district hospital which receives many high-risk pregnancies referred from 12 health centers of which some are preterm babies. It has a busier maternity department and registers about 300 births every month. It equally has a busy neonatal unit that provides specialized neonatal care (SNC) services. The hospital SNC is comprised of medical staffs: paediatricians (2), medical officers (2) and 12 nurses (12). Kabutare district hospital is located in Huye district, southern province of Rwanda, at 126 km from the capital Kigali. Huye district has a population of 328,605 inhabitants covering an area of 581.5 km^2^. Its density population is 510.3 inhabitants per km^2^.

**Study population:** the study population comprised of postnatal women who delivered preterm babies between August and September 2020. The sample size was determined using single population proportion formula by considering confidence level (95%), margin of error = 0.04 and proportion of preterm rate in Rwanda being 10% [7]. By adding 10% non-response rate, the final sample size was 240.

**Inclusion/exclusion criteria:** consenting postnatal women aged between 18 and 49 years who had delivered a singleton preterm baby within the study period were included. On the other hand, non-consenting postnatal women, mentally unstable, those who had memory loss or those who were sick thus unable to participate were excluded from the study.

**Study variables:** the dependent variable was spontaneous preterm birth. The independent variables were as follows: (i) maternal socio-demographic characteristics (age, residence, marital status, religion, level of education, family size, occupation and Mid-Upper Arm Circumference [MUAC]); (ii) maternal pregnancy lifestyle factors (smoking during pregnancy, alcohol consumption, husband being cigarette smoker, husband drinks alcohol; (iii) antenatal care (ANC)-related factors (frequency of ANC visits, gestational age of first visit, prior pregnancy danger signs and maternal HIV status) and obstetric-related factors (parity, inter-delivery interval, onset of labour, gestational age, previous preterm birth, anaemia during pregnancy).

**Operational definitions:** preterm birth was the commencement of labor with an intact or pre-labor rapture of the membrane and birth before 37 weeks of gestational age. The inter-delivery was defined as the number of months since the previous birth. It was considered as either being short or optimal if the birth interval was ≤ 24 months or ≥ 25 months, respectively.

**Study procedure:** we conducted a systematic sampling which enabled us recruit all mothers who had delivered within 24 hours at Kabutare hospital from August to September 2020. Informed consent was obtained from the mothers whose babies had been admitted to the newborn care unit (NBU). A semi-structured pre-test questionnaire was administered to the mothers while additional data were obtained from the mothers' and babies' obstetric and neonatal records for those admitted as required, respectively. Data were collected from medical records by two trained research assistants who were supervised by the principal investigator. For every case, information was collected regarding socio-demographic characteristics, medical history, antenatal care attendance (ANC), medical conditions diagnosed before or during current pregnancy and details of lifestyle and anthropometric measurements and maternal and perinatal outcome including complications. Maternal nutritional status was assessed by measuring the left mid-upper arm circumference (MUAC) using non-stretchable MUAC tapes used for screening pregnant mothers. A low MUAC was defined as a measurement of less than 23 cm. While, gestational age was calculated using last menstrual period and confirmed within 24 hours of birth by clinical assessment using the Ballard Score.

**Ethical considerations:** ethical approval was obtained from Mount Kenya University Kigali, Rwanda The School of Postgraduate studies, Mount Kenya University, Kigali first approved the research proposal and ethical clearance was obtained from the Institutional Research and Ethics committee of Mount Kenya University (N°: MKU04/DVCARA/2019-2020/077). After obtaining ethical approval, permission was obtained from the director general of Kabutare district hospital for authorization to proceed with the data collection. The participants were told about the study objectives and that their participation was voluntary and they could withdraw at any time, without giving any reason. Written informed consent was obtained for participation in the study. No inducements or rewards were given to participants to join the study. Confidentiality was maintained at all times. Data collected as part of the study were not linked to individual or personal identifiers and was reported in accordance with the STROBE guidelines.

**Data analysis:** data were entered into Microsoft Access database, cleaned and uploaded into a password protected android tablet. The outcome variables were dichotomized and coded as 0 and 1, representing those that did not have a preterm birth and those that had a preterm birth, respectively. Data were analyzed using Stata version 17 Univariate analysis was done to include frequencies and proportions that was displayed in form of tables and figures. Bivariate analysis using Chi-square test was conducted to identify the association between the independent and dependent variables. Adjusted Odds Ratios, and their 95% confidence intervals were deliberated for multivariable logistic regression analysis to take care of all potential confounders. The goodness of fit was assessed using Hosmer-Lemeshow test with Chi-square value 1.74 and p-value of 0.884 which indicated that the fitted model was adequate.

## Results

**Socio-demographic characteristics:** of all 240 women who were interviewed, mean age was 29 years. Maternal socio-demographic characteristics are shown in Table 1 and there was significant difference between maternal MUAC measurement and preterm birth where neonates whose mothers had MUAC < 23cm were more likely to be delivered with premature birth (P <0.001).

**Table 1:** maternal socio-demographic characteristics by preterm birth

Variables	Total, n (%)	Pre-term		Chi-square value	P
		Yes, n (%)	No, n (%)		
**Age in years**					
< 19	18 (7.5)	3 (7.1)	15 (7.6)	0.12	0.944
20 - 34	164 (68.5)	28 (66.7)	136 (68.7)		
≥ 35	58 (24.2)	11 (26.2)	47 (23.7)		
**Residence**					
Urban	25 (10.4)	5 (11.9)	20 (10.1)	0.12	0.728
Rural	215 (89.6)	37 (88.9)	178 (89.9)		
**Marital status**					
Married	176 (73.3)	32 (76.2)	144 (72.7)	0.21	0.645
Single/divorced/widowed	64 (26.7)	10 (23.8)	54 (27.3)		
**Religion**					
Protestant	228 (95.0)	41 (97.6)	187 (94.4)	0.74	0.391
Muslim	12 (5.0)	1 (2.4)	11 (5.6)		
**Level of education**					
Primary	236 (98.3)	41 (97.6)	195 (98.5)	0.16	0.691
Secondary	4 (1.7)	1 (2.4)	3 (1.5)		
**Family size**					
<3	57 (23.8)	7 (16.7)	50 (25.3)	1.64	0.651
3 to 4	107 (44.6)	20 (47.6)	87 (43.9)		
5 to 6	54 (22.5)	10 (23.8)	44 (22.2)		
>6	22 (9.2)	5 (11.9)	17 (8.6)		
**Occupation**					
Farmer	225 (93.8)	41 (97.6)	184 (92.9)	1.61	0.447
Small business running	9 (3.8)	1 (2.4)	8 (4.0)		
Others	6 (2.5)	0 (0.0)	6 (3.1)		
**MUAC (cm)**					
<23	44 (18.3)	17 (40.5)	27 (13.6)	16.67	<0.001*
≥ 23	196 (81.7)	25 (59.5)	171 (86.4)		

**Prevalence of preterm birth:** out of 240 women who delivered in Kabutare hospital, 42 babies were born as preterm giving prevalence of 17.5% (95% CI: 12.9% - 22.9%).

**Pregnancy lifestyle factors:** compared to women who delivered at term, women who delivered preterm when pregnant lived lifestyles associated with cigarette smoking, drinking alcohol and passive smoking (Table 2).

**Table 2:** pregnancy lifestyle factors by preterm birth

Variables	Total, n (%)	Pre-term		Chi-square value	P
		Yes, n (%)	No, n (%)		
**Maternal smoking during pregnancy**					
Yes	13 (5.4)	6 (14.3)	7 (3.5)	7.816	0.005*
No	227 (94.6)	36 (85.7)	191 (96.5)		
**Maternal alcohol consumption**					
Yes	56 (23.3)	15 (35.7)	41 (20.7)	4.362	0.037*
No	184 (76.7)	27 (64.3)	157 (79.3)		
**Husband being a cigarette smoker**					
Yes	48 (20.0)	18 (42.9)	30 (15.2)	16.623	<0.001*
No	192 (80.0)	24 (57.1)	168 (84.8)		
**Husband drinks alcohol**					
Yes	155 (64.6)	28 (66.6)	127 (64.1)	0.097	0.756
No	85 (35.4)	14 (33.3)	71 (35.9)		

**Antenatal and obstetric factors:** regarding antenatal and obstetric factors, mothers who had ANC attendance ≤ 3 times and prior experience of pregnancy danger sign were likely to deliver preterm. Whereas, aneamia during pregnancy, inter-delivery interval and prior preterm delivery were not significant (Table 3).

**Table 3:** antenatal care and obstetric factors associated with preterm birth

Variables	Total, n (%)	Pre-term		Chi-square value	P
		Yes, n (%)	No, n (%)		
**Parity**					
1	95 (39.6)	14 (33.3)	81 (40.9)	1.30	0.599
2 to 4	118 (49.2)	22 (52.4)	96 (48.5)		
≥ 5	27 (11.3)	6 (14.3)	21 (10.6)		
**Inter-delivery interval** (months)					
≤ 24	16 (6.7)	6 (14.3)	10 (5.1)	4.76	0.093
≥ 25	67 (27.9)	11 (26.2)	56 (28.2)		
Don't know	157 (65.4)	25 (59.5)	132 (66.7)		
**Antenatal attendance**					
≤ 3	142 (59.2)	37 (88.1)	105 (53.0)	17.63	<0.001*
≥ 4	98 (40.8)	5 (11.9)	93 (47.0)		
**Gestational age of first ANC visit** (weeks)					
< 12	156 (65.0)	29 (69.0)	127 (64.1)	1.23	0.541
13- 18	61 (25.4)	8 (19.0)	53 (26.8)		
≥ 19	23 (9.6)	5 (12.0)	18 (9.1)		
**Prior pregnancy danger signs**					
Yes	17 (7.1)	7 (16.7)	10 (5.0)	7.10	0.008*
No	223 (92.9)	35 (83.3)	188 (95.0)		
**Maternal HIV status**					
Negative	229 (95.4)	41 (97.6)	188 (82.1)	0.57	0.452
Positive	11 (4.6)	1 (2.4)	10 (90.9)		
**Previous preterm birth**					
Yes	7 (2.9)	4 (9.5)	3 (1.5)	1.96	0.374
No	166 (69.2)	33 (78.6)	133 (67.2)		
Don’t know	67 (27.9)	5 (11.9)	62 (31.3)		
**Anaemia during Pregnancy (**Hemoglobin level gm/dL)					
Low (<10)	4 (1.7)	1 (5.0)	3 (1.5)	0.16	0.691
Normal (≥11)	236 (98.3)	41 (95.0)	195 (98.5)		

**Factors associated with preterm birth:** the adjusted multivariate model showed that significant associated factors with preterm birth included: husband being a smoker (adjusted Odds Ratio (aOR) = 5.9; 95% CI; 1.9-18; p= 0.002), ANC attendance ≤ 3 visits (aOR=3.9; 95% CI; 1.1-13.8; p=0.04) and mother's MUAC < 23cm (aOR=5.6, 95% CI; 1.8-18.9; p=0.004) (Table 4).

**Table 4:** factors associated with of preterm delivery

Variables	AOR	95%CI		P
		Lower	Upper	
**Frequency of ANC visits**				
≤ 3	3.87	1.09	13.8	0.037*
≥ 4	Ref			
**Danger signs during ANC visits**				
Yes	1.82	0.35	9.52	0.476
No	Ref			
**Cigarette consumption during pregnancy**				
Yes	0.88	0.18	4.34	0.87
No	Ref			
**Alcohol drinking during pregnancy**				
Yes	1.66	0.56	4.92	0.362
No	Ref			
Cigarette consumption by husband during pregnancy				
Yes	5.92	1.94	18.04	0.002*
No	Ref			
**MUAC (cm)**				
< 23	5.75	1.75	18.87	0.004*
≥ 23	Ref			

AOR= Adjusted Odds Ratio; CI= Confidence Interval, Ref: Reference; *P < 0.05.

## Discussion

Our results add importantly to the literature about the prevalence of preterm birth from remote hospital setting [3]. Our findings revealed that the prevalence of preterm birth among neonates was 17.5 per cent livebirths. Husband being smoker, ANC attendance ≤ 3 times and mother's MUAC < 23cm were predictors of premature delivery.

The preterm rate for this study almost doubled the WHO estimates of 9.5% for sub-Saharan Africa [10] as well more than the reported incidence of preterm birth in Rwanda [9,11] Kenya [12] and Tanzania [13]. This high prevalence may be due to the fact that it is policy to refer all high-risk cases. This discrepancy could be because our study was conducted from a district hospital whereby most pregnant mothers were referred from lower health centers with complex obstetric and gynecological issues. In addition, Ultrasound is one of the confirmatory diagnostic criteria for preterm birth but not done in our study, we used last menstrual period and Ballard score which might lead to imprecise gestational age estimation. So, this self-reported of last menstrual period may give high or low estimation of preterm birth.

Our findings revealed that mothers who attended less than 4 times of WHO recommended ANC visits had 4-fold higher probability to deliver a preterm baby. This agrees with a meta-analysis and systematic review [14]. In addition, this finding concurs with various studies done in northern Ethiopia [15], Tanzania [13], south-western Nigeria [16] and western China [17]. This signifies that adequate and focused ANC is paramount to prevent preterm birth. This is evident from our study whereby anaemia during pregnancy and maternal HIV status was not associated with preterm delivery as all pregnant mothers for came for ANC receive iron and folate supplements as early as possible [18] as well as easily accessibility and use of antiretroviral drugs for prophylaxis and treatment of HIV among pregnant women in Rwanda [19].

Paternal smoking was independently associated with preterm births where neonates whose fathers were smoking were six times more likely to have prematurity. It is evidenced that a lot of chemicals including nicotine can be passed to the fetus through passive smoking to pregnant women [20] thus, adverse effects inclusive premature birth. In a recent systematic review and meta-analysis indicated that parental smoking was connected with high chance of premature births. However, some studies did not find a significant association between paternal smoking and premature birth [21]. Therefore, preventive strategies are of utmost importance, such as educating pregnant women during ANC on need to change their usual lifestyles through omitting behaviours such as alcohol consumption, smoking inclusive passive smoking.

Our findings revealed that a low maternal MUAC was six times more associated with preterm birth. This was in agreement with authors from Ethiopia [22] and Bangladesh [23]. As indicated in other studies, mother's undernutrition is related with an upsurge chance of morbidity and mortality with a number of bad pregnancy outcomes like low birth weight and premature delivery as consequence to bad effect on placental size, embryo and quality of membrane [24]. Our findings as well as exiting latter evidence aids a secure relationship with mother's undernutrition with poor pregnancy and delivery outcomes like premature delivery and low birth weight. Thus, reinforces national programs to setup mediation plans centering on intensifying nutritional status of mothers in reproductive age and in the gestation period which would assist in attaining the above-mentioned targets of 2030.

Maternal smoking and liquor drinking during pregnancy were significantly connected with premature birth during bivariate analysis but was insignificant after multivariable analysis. However, there is evidence that smoking during pregnancy may pass nicotine to the fetus through placental barriers and it is 15% more concentration present in placenta than maternal blood. The risk of premature delivery is high amid those mothers that are active smokers during pregnancy [25]. However, in this study the proportion of women smoking was small and most among the smokers were smoking occasionally. Furthermore, many studies have demonstrated that light to moderate liquor drinking during pregnancy could not significantly affect premature birth [6]. However, heavy liquor drinking especially in second and third trimester has a significant risk of preterm delivery [26]. Besides, different studies using systematic review on alcohol drinking have shown protective or risk for preterm delivery [27].

The strength of this study is that mothers who had live births were shortly interviewed after birth and babies their assessed for gestational age, minimizing recall bias. Moreover, some women may recall or provide information about preterm birth selectively, depending on their experience during birth or pregnancy outcome. In terms of limitations, the nature of study design being cross-sectional may not show the cause-effect relationship. We used last menstrual period to determine preterm birth with and clinical assessment of gestation using the Ballard score might lead to over/under estimate of the burden. Use of secondary data for some variables is another limitation of our study. There is need for a cohort study to further investigate the effect of paternal smoking on preterm birth.

## Conclusion

The prevalence of preterm birth at Kabutare district hospital was 17.5%. Husband being smoker, ANC attendance ≤ 3 times and low mother's MUAC < 23cm were notably associated with preterm delivery. We recommend that ANC sessions to re-emphasize on maternal nutritional education/counseling which is of good quality and quantity, discourage maternal alcohol consumption as well as passive smoking. Policymakers should extend screening and nutritional programs of pregnant mothers within antenatal care package.

### What is known about this topic

- *Globally, the leading cause of neonatal mortality in preterm birth which may hinder the achievement of sustainable development goals 3.2 target;*
- *In Rwanda, 43,000 babies are born too soon each year; out of that, 2,070 die due to direct preterm complications which explains why there was a slight decline in neonatal mortality rate from 20 to 19 per 1,000 livebirth in 2015 and 2020, respectively;*
- *It is well known that the degree of prematurity inversely relates to the likelihood of increased mortality and morbidity*.

### What this study adds

- *One out of five babies were born as preterm in a rural Rwandan district hospital;*
- *Having a husband who smokes cigarettes, attendance of less than three antenatal care visits and maternal undernutrition were notably associated with preterm delivery;*
- *To reduce this burden of prematurity, antenatal care providers should re-emphasize on the importance of pregnant women nutritional screening and education/counselling which is of good quality and quantity, discourage maternal alcohol consumption as well as passive smoking*.
